# Supplementary material for: Modeling glioblastoma heterogeneity as a dynamic network of cell states
Source: Mol Syst Biol. 2021 Sep 16;17(9):e10105. doi: 10.15252/msb.202010105 (PMC8444284; doi:10.15252/msb.202010105)
Supplement: Supplementary file 5 — Source Data for Figure 3 [file MSB-17-e10105-s001.zip › Figure3A_sourcedata/GSEA_3065/hallmarks_state3.GseaPreranked.1623416274744/HALLMARK_MITOTIC_SPINDLE.html]

Details for gene set HALLMARK\_MITOTIC\_SPINDLE[GSEA]

|  || Dataset | state3 |
| Phenotype | NoPhenotypeAvailable |
| Upregulated in class | na\_pos |
| GeneSet | HALLMARK\_MITOTIC\_SPINDLE |
| Enrichment Score (ES) | 0.48370782 |
| Normalized Enrichment Score (NES) | 2.054247 |
| Nominal p-value | 0.0 |
| FDR q-value | 0.0 |
| FWER p-Value | 0.0 |
Table: GSEA Results Summary

  

Fig 1: Enrichment plot: HALLMARK\_MITOTIC\_SPINDLE      
 Profile of the Running ES Score & Positions of GeneSet Members on the Rank Ordered List

  

| PROBE | GENE SYMBOL | GENE\_TITLE | RANK IN GENE LIST | RANK METRIC SCORE | RUNNING ES | CORE ENRICHMENT || 1 | CENPF |  |  | 4 | 0.619 | 0.0334 | Yes |
| 2 | TOP2A |  |  | 42 | 0.448 | 0.0537 | Yes |
| 3 | DYNC1H1 |  |  | 47 | 0.437 | 0.0771 | Yes |
| 4 | SPTBN1 |  |  | 61 | 0.394 | 0.0972 | Yes |
| 5 | PIF1 |  |  | 64 | 0.389 | 0.1182 | Yes |
| 6 | MYH10 |  |  | 79 | 0.366 | 0.1366 | Yes |
| 7 | DST |  |  | 80 | 0.365 | 0.1566 | Yes |
| 8 | CENPE |  |  | 126 | 0.321 | 0.1689 | Yes |
| 9 | DLGAP5 |  |  | 207 | 0.279 | 0.1748 | Yes |
| 10 | SMC1A |  |  | 231 | 0.271 | 0.1870 | Yes |
| 11 | TRIO |  |  | 235 | 0.269 | 0.2014 | Yes |
| 12 | SMC4 |  |  | 265 | 0.255 | 0.2120 | Yes |
| 13 | ANLN |  |  | 281 | 0.249 | 0.2239 | Yes |
| 14 | PCM1 |  |  | 325 | 0.237 | 0.2319 | Yes |
| 15 | SMC3 |  |  | 341 | 0.231 | 0.2428 | Yes |
| 16 | CKAP5 |  |  | 366 | 0.227 | 0.2524 | Yes |
| 17 | SPTAN1 |  |  | 376 | 0.223 | 0.2636 | Yes |
| 18 | KIF20B |  |  | 411 | 0.216 | 0.2714 | Yes |
| 19 | NF1 |  |  | 425 | 0.214 | 0.2816 | Yes |
| 20 | KIF15 |  |  | 428 | 0.213 | 0.2930 | Yes |
| 21 | ACTN4 |  |  | 449 | 0.210 | 0.3022 | Yes |
| 22 | KIF11 |  |  | 451 | 0.209 | 0.3135 | Yes |
| 23 | AKAP13 |  |  | 452 | 0.209 | 0.3250 | Yes |
| 24 | ECT2 |  |  | 527 | 0.195 | 0.3270 | Yes |
| 25 | TPX2 |  |  | 544 | 0.193 | 0.3357 | Yes |
| 26 | EPB41L2 |  |  | 596 | 0.187 | 0.3400 | Yes |
| 27 | PCNT |  |  | 615 | 0.184 | 0.3480 | Yes |
| 28 | APC |  |  | 616 | 0.184 | 0.3581 | Yes |
| 29 | FLNA |  |  | 633 | 0.181 | 0.3661 | Yes |
| 30 | KIF23 |  |  | 662 | 0.177 | 0.3725 | Yes |
| 31 | MYH9 |  |  | 688 | 0.173 | 0.3791 | Yes |
| 32 | CDK5RAP2 |  |  | 699 | 0.170 | 0.3872 | Yes |
| 33 | CEP192 |  |  | 710 | 0.169 | 0.3953 | Yes |
| 34 | BUB1 |  |  | 717 | 0.168 | 0.4038 | Yes |
| 35 | LRPPRC |  |  | 754 | 0.163 | 0.4086 | Yes |
| 36 | CNTRL |  |  | 756 | 0.163 | 0.4174 | Yes |
| 37 | EZR |  |  | 759 | 0.162 | 0.4260 | Yes |
| 38 | KIF1B |  |  | 767 | 0.161 | 0.4340 | Yes |
| 39 | ARFGEF1 |  |  | 815 | 0.156 | 0.4371 | Yes |
| 40 | VCL |  |  | 834 | 0.154 | 0.4434 | Yes |
| 41 | CDK1 |  |  | 915 | 0.147 | 0.4422 | Yes |
| 42 | NIN |  |  | 933 | 0.145 | 0.4481 | Yes |
| 43 | KNTC1 |  |  | 935 | 0.144 | 0.4559 | Yes |
| 44 | LMNB1 |  |  | 1021 | 0.137 | 0.4535 | Yes |
| 45 | NDC80 |  |  | 1087 | 0.131 | 0.4531 | Yes |
| 46 | BRCA2 |  |  | 1122 | 0.128 | 0.4561 | Yes |
| 47 | CDC42BPA |  |  | 1141 | 0.127 | 0.4610 | Yes |
| 48 | RAB3GAP1 |  |  | 1175 | 0.123 | 0.4639 | Yes |
| 49 | TTK |  |  | 1177 | 0.123 | 0.4705 | Yes |
| 50 | CCDC88A |  |  | 1249 | 0.118 | 0.4687 | Yes |
| 51 | SUN2 |  |  | 1275 | 0.117 | 0.4722 | Yes |
| 52 | ARHGEF2 |  |  | 1323 | 0.114 | 0.4729 | Yes |
| 53 | FLNB |  |  | 1335 | 0.113 | 0.4778 | Yes |
| 54 | NUSAP1 |  |  | 1338 | 0.113 | 0.4837 | Yes |
| 55 | CEP250 |  |  | 1439 | 0.106 | 0.4779 | No |
| 56 | CENPJ |  |  | 1459 | 0.105 | 0.4814 | No |
| 57 | EPB41 |  |  | 1504 | 0.103 | 0.4819 | No |
| 58 | MYO9B |  |  | 1590 | 0.098 | 0.4774 | No |
| 59 | ROCK1 |  |  | 1665 | 0.093 | 0.4738 | No |
| 60 | CLIP1 |  |  | 1743 | 0.089 | 0.4697 | No |
| 61 | RICTOR |  |  | 1760 | 0.088 | 0.4727 | No |
| 62 | OPHN1 |  |  | 1858 | 0.083 | 0.4660 | No |
| 63 | TUBGCP5 |  |  | 1860 | 0.083 | 0.4704 | No |
| 64 | RHOT2 |  |  | 1876 | 0.082 | 0.4731 | No |
| 65 | HOOK3 |  |  | 1934 | 0.079 | 0.4708 | No |
| 66 | KIF2C |  |  | 1985 | 0.077 | 0.4692 | No |
| 67 | NOTCH2 |  |  | 1990 | 0.076 | 0.4729 | No |
| 68 | NCK1 |  |  | 1998 | 0.076 | 0.4762 | No |
| 69 | HDAC6 |  |  | 2080 | 0.073 | 0.4708 | No |
| 70 | TSC1 |  |  | 2123 | 0.071 | 0.4698 | No |
| 71 | MAP1S |  |  | 2207 | 0.068 | 0.4638 | No |
| 72 | CTTN |  |  | 2280 | 0.065 | 0.4590 | No |
| 73 | RFC1 |  |  | 2360 | 0.062 | 0.4532 | No |
| 74 | TUBGCP3 |  |  | 2468 | 0.058 | 0.4439 | No |
| 75 | DLG1 |  |  | 2526 | 0.056 | 0.4403 | No |
| 76 | KIF22 |  |  | 2545 | 0.055 | 0.4413 | No |
| 77 | TUBGCP6 |  |  | 2613 | 0.053 | 0.4363 | No |
| 78 | CSNK1D |  |  | 2754 | 0.049 | 0.4227 | No |
| 79 | RACGAP1 |  |  | 2769 | 0.048 | 0.4237 | No |
| 80 | ARHGEF7 |  |  | 2867 | 0.045 | 0.4149 | No |
| 81 | PLEKHG2 |  |  | 2889 | 0.044 | 0.4148 | No |
| 82 | CEP57 |  |  | 2970 | 0.042 | 0.4078 | No |
| 83 | ARHGEF11 |  |  | 3058 | 0.040 | 0.3998 | No |
| 84 | KIF4A |  |  | 3195 | 0.036 | 0.3859 | No |
| 85 | PKD2 |  |  | 3227 | 0.035 | 0.3842 | No |
| 86 | NUMA1 |  |  | 3232 | 0.035 | 0.3857 | No |
| 87 | NCK2 |  |  | 3255 | 0.034 | 0.3850 | No |
| 88 | RABGAP1 |  |  | 3272 | 0.034 | 0.3850 | No |
| 89 | TLK1 |  |  | 3326 | 0.032 | 0.3806 | No |
| 90 | SSH2 |  |  | 3405 | 0.030 | 0.3732 | No |
| 91 | CCNB2 |  |  | 3502 | 0.028 | 0.3635 | No |
| 92 | AURKA |  |  | 3503 | 0.028 | 0.3650 | No |
| 93 | CDC27 |  |  | 3508 | 0.028 | 0.3661 | No |
| 94 | STK38L |  |  | 3574 | 0.026 | 0.3600 | No |
| 95 | FARP1 |  |  | 3580 | 0.026 | 0.3608 | No |
| 96 | TBCD |  |  | 3615 | 0.025 | 0.3582 | No |
| 97 | RASAL2 |  |  | 3679 | 0.023 | 0.3522 | No |
| 98 | ALMS1 |  |  | 3716 | 0.022 | 0.3492 | No |
| 99 | ARHGAP5 |  |  | 3722 | 0.022 | 0.3498 | No |
| 100 | ABL1 |  |  | 3737 | 0.022 | 0.3494 | No |
| 101 | MAP3K11 |  |  | 3763 | 0.021 | 0.3476 | No |
| 102 | SASS6 |  |  | 3780 | 0.021 | 0.3469 | No |
| 103 | ABR |  |  | 3796 | 0.020 | 0.3462 | No |
| 104 | PRC1 |  |  | 3878 | 0.018 | 0.3378 | No |
| 105 | TUBGCP2 |  |  | 3882 | 0.018 | 0.3384 | No |
| 106 | KLC1 |  |  | 3891 | 0.018 | 0.3384 | No |
| 107 | ALS2 |  |  | 3990 | 0.015 | 0.3279 | No |
| 108 | GSN |  |  | 4064 | 0.013 | 0.3201 | No |
| 109 | ARHGAP10 |  |  | 4118 | 0.012 | 0.3146 | No |
| 110 | FBXO5 |  |  | 4119 | 0.012 | 0.3152 | No |
| 111 | KIF5B |  |  | 4156 | 0.011 | 0.3116 | No |
| 112 | CEP131 |  |  | 4269 | 0.008 | 0.2990 | No |
| 113 | PDLIM5 |  |  | 4368 | 0.005 | 0.2878 | No |
| 114 | ARHGEF12 |  |  | 4401 | 0.004 | 0.2843 | No |
| 115 | CLASP1 |  |  | 4435 | 0.003 | 0.2806 | No |
| 116 | RASA1 |  |  | 4447 | 0.003 | 0.2795 | No |
| 117 | RASA2 |  |  | 4517 | 0.001 | 0.2715 | No |
| 118 | MID1 |  |  | 4556 | -0.000 | 0.2671 | No |
| 119 | GEMIN4 |  |  | 4648 | -0.002 | 0.2566 | No |
| 120 | LATS1 |  |  | 4964 | -0.010 | 0.2204 | No |
| 121 | KIF3C |  |  | 5019 | -0.012 | 0.2148 | No |
| 122 | PAFAH1B1 |  |  | 5051 | -0.012 | 0.2118 | No |
| 123 | SOS1 |  |  | 5067 | -0.013 | 0.2107 | No |
| 124 | MARK4 |  |  | 5076 | -0.013 | 0.2105 | No |
| 125 | CYTH2 |  |  | 5085 | -0.013 | 0.2103 | No |
| 126 | ARL8A |  |  | 5093 | -0.013 | 0.2102 | No |
| 127 | BCR |  |  | 5096 | -0.013 | 0.2107 | No |
| 128 | TAOK2 |  |  | 5207 | -0.016 | 0.1988 | No |
| 129 | PLK1 |  |  | 5235 | -0.017 | 0.1965 | No |
| 130 | FGD4 |  |  | 5246 | -0.017 | 0.1963 | No |
| 131 | KIFAP3 |  |  | 5280 | -0.018 | 0.1934 | No |
| 132 | PREX1 |  |  | 5327 | -0.019 | 0.1891 | No |
| 133 | ARFIP2 |  |  | 5377 | -0.020 | 0.1845 | No |
| 134 | RANBP9 |  |  | 5385 | -0.021 | 0.1848 | No |
| 135 | ITSN1 |  |  | 5427 | -0.022 | 0.1812 | No |
| 136 | NEDD9 |  |  | 5524 | -0.025 | 0.1714 | No |
| 137 | ATG4B |  |  | 5660 | -0.029 | 0.1572 | No |
| 138 | ABI1 |  |  | 5708 | -0.030 | 0.1534 | No |
| 139 | TIAM1 |  |  | 5861 | -0.035 | 0.1376 | No |
| 140 | DYNLL2 |  |  | 5969 | -0.038 | 0.1272 | No |
| 141 | CDC42EP4 |  |  | 6004 | -0.040 | 0.1254 | No |
| 142 | WASF1 |  |  | 6025 | -0.040 | 0.1253 | No |
| 143 | CLIP2 |  |  | 6201 | -0.046 | 0.1074 | No |
| 144 | ARHGEF3 |  |  | 6239 | -0.048 | 0.1057 | No |
| 145 | KATNA1 |  |  | 6314 | -0.050 | 0.0998 | No |
| 146 | CD2AP |  |  | 6408 | -0.053 | 0.0919 | No |
| 147 | KIF3B |  |  | 6471 | -0.055 | 0.0877 | No |
| 148 | SHROOM2 |  |  | 6544 | -0.058 | 0.0825 | No |
| 149 | ARHGAP29 |  |  | 6596 | -0.060 | 0.0798 | No |
| 150 | MID1IP1 |  |  | 6625 | -0.062 | 0.0800 | No |
| 151 | SAC3D1 |  |  | 6692 | -0.065 | 0.0759 | No |
| 152 | MAPRE1 |  |  | 6891 | -0.075 | 0.0569 | No |
| 153 | PALLD |  |  | 6929 | -0.077 | 0.0568 | No |
| 154 | SORBS2 |  |  | 6955 | -0.078 | 0.0582 | No |
| 155 | CDC42 |  |  | 7126 | -0.089 | 0.0432 | No |
| 156 | WASL |  |  | 7134 | -0.089 | 0.0473 | No |
| 157 | CDC42EP1 |  |  | 7138 | -0.090 | 0.0519 | No |
| 158 | BCAR1 |  |  | 7232 | -0.096 | 0.0463 | No |
| 159 | WASF2 |  |  | 7236 | -0.096 | 0.0512 | No |
| 160 | KATNB1 |  |  | 7293 | -0.100 | 0.0501 | No |
| 161 | PPP4R2 |  |  | 7306 | -0.101 | 0.0543 | No |
| 162 | PXN |  |  | 7344 | -0.104 | 0.0556 | No |
| 163 | BIN1 |  |  | 7531 | -0.120 | 0.0405 | No |
| 164 | ARF6 |  |  | 7536 | -0.121 | 0.0467 | No |
| 165 | YWHAE |  |  | 7628 | -0.131 | 0.0432 | No |
| 166 | PCGF5 |  |  | 7719 | -0.142 | 0.0405 | No |
| 167 | BIRC5 |  |  | 7735 | -0.144 | 0.0466 | No |
| 168 | STAU1 |  |  | 7955 | -0.178 | 0.0309 | No |
| 169 | RALBP1 |  |  | 8016 | -0.188 | 0.0342 | No |
| 170 | FSCN1 |  |  | 8144 | -0.214 | 0.0311 | No |
| 171 | UXT |  |  | 8163 | -0.219 | 0.0409 | No |
| 172 | ARHGDIA |  |  | 8218 | -0.233 | 0.0474 | No |
| 173 | CAPZB |  |  | 8336 | -0.267 | 0.0484 | No |
Table: GSEA details [plain text format]

  

Fig 2: HALLMARK\_MITOTIC\_SPINDLE: Random ES distribution      
 Gene set null distribution of ES for **HALLMARK\_MITOTIC\_SPINDLE**
